# Recent Advances in Pediatric Otolaryngology

**DOI:** 10.1155/2012/535016

**Published:** 2012-06-06

**Authors:** Jeffrey A. Koempel, Tomislav Baudoin, Alan T. L. Cheng, Debra M. Don, Ajoy M. Varghese

**Affiliations:** ^1^Division of Otolaryngology-Head and Neck Surgery, Children's Hospital, Los Angeles, CA 90027, USA; ^2^Department of Otolaryngology-Head and Neck Surgery, Keck School of Medicine of the University of Southern California, Los Angeles, CA 90089, USA; ^3^Referral Center for Pediatric Otolaryngology, Sisters of Charity University Hospital, Ministry of Health and Social Welfare of the Republic of Croatia, 10000 Zagreb, Croatia; ^4^Department of Pediatric Otolaryngology, The Sydney Children's Hospital Network—Westmead Campus, The University of Sydney, Sydney NSW 2145, Australia; ^5^Department of ENT-2, Christian Medical College, Vellore 632004, India

Ear, nose, and throat problems comprise a significant portion of patient visits to the primary care physicians' offices, urgent care facilities, emergency rooms, and children's hospitals. Since its beginnings in the 1970s, the specialty of pediatric otolaryngology has developed significantly and even more so in the last five to ten years. The aim of this special issue is to offer our pediatrician colleagues an opportunity to learn about recent advances in both diagnostic methods and therapeutic procedures that are now available to assist in the care of children with ear, nose, and throat disorders. 

This special issue is comprised of both original clinical research and review articles from all areas of pediatric otolaryngology such as otologic disease and hearing loss, sinonasal disorders, airway issues, and head and neck masses. These papers emphasize how methods of diagnosis have improved for certain conditions, how existing surgical procedures have been modified to be less invasive and better tolerated, and finally, how new technology and operations allow clinicians to treat otolaryngologic disorders that were without treatment in the past.

The paper “*Troublesome tinnitus in children: epidemiology, audiological profile, and preliminary Results of Treatment*” by G. Bartnik et al. offers a very readable and practical approach to a common otologic problem in children while most pediatric textbooks often have no information on this subject. This group offers their experience with a treatment called “Tinnitus Retraining Therapy.” A. Koravand, B. Jutras, and M. Lassonde present their original research using the diagnostic tools of cortical auditory evoked potentials and mismatch responses (MMRs) to identify patterns of neural activity in the central auditory system of children with hearing loss.

The care of patients with nose and sinus complaints can be frustrating to the primary care physician. H. Ramadan's paper “*Chronic rhinosinusitis in children*” provides a simple, straightforward approach to the pediatric patient with these symptoms including the use of “real-life” examples to illustrate the important aspects of the history and physical exam that are necessary in the care of these patients. After reading this article, any health care practitioner will feel more confident about their decision-making in patients with sinus disease. Although juvenile angiofibroma is a rare clinical problem, P. Nicolai, A. Schreiber, and A. B. Villaret describe new, less invasive approaches to treatment of this sinonasal mass. This is in addition to a succinct review of the pathophysiology and recommended work-up including the relative advantages and disadvantages of various radiographic imaging techniques.

The airway section of this special issue will be of great value to the primary care practitioner. Included are three papers on stridor in the infant, one dedicated entirely to laryngomalacia, and lastly, a paper on a new diagnostic tool called “sleep endoscopy.” M. Daniel and A. Cheng discuss the approach to an infant with noisy breathing or stridor; separating the material into three sections that allow for easy recall and use by the pediatrician. Novel approaches such as the EXIT procedure to treat disorders of the fetal airway are presented. A. M. Landry and D. M. Thompson's review article on laryngomalacia will be equally relevant. The reader will find it to be complete yet easily digestible. Finally, any health care practitioner will be familiar with the frustration of seeing patients for persistent difficulty breathing at night after adenotonsillectomy for upper airway obstruction or obstructive sleep apnea. Although polysomnography can determine the presence and severity of any degree of obstruction, it cannot identify the location of obstruction. The paper by A. C. Lin and P. J. Koltai describe a new diagnostic tool called “sleep endoscopy” that will allow otolaryngologists the opportunity to identify such areas of obstruction and perhaps offer treatment options which were not obvious in the past.

The approach to a patient with a vascular malformation can be confusing. G. T. Richter's review article on this clinical entity will be very helpful to the primary care practitioner. Nomenclature, relevant parts of the history and physical exam, diagnostic tools including radiographic imaging studies, and medical and surgical therapies are discussed. D. Dzepina's paper on papillary thyroid carcinoma in children describes their approach and results with total thyroidectomy including neck dissection for lymphatic dissemination which is common in this type of thyroid cancer.

The editors of this special issue have worked hard with the authors to provide primary care practitioners with information that is relevant to their practice and, at the same time, very easy to read and understand. We hope the papers in this special issue will be of great help to primary care practitioners as they see pediatric patients with ear, nose, and throat problems.

*Jeffrey A. Koempel* *Jeffrey A. Koempel* *Tomislav Baudoin* *Tomislav Baudoin* *Alan T. L. Cheng* *Alan T. L. Cheng* *Debra M. Don* *Debra M. Don* *Ajoy M. Varghese* *Ajoy M. Varghese*

